# mRNA expression of the severe acute respiratory syndrome-coronavirus 2 angiotensin-converting enzyme 2 receptor in the lung tissue of Wistar rats according to age

**DOI:** 10.14202/vetworld.2022.427-434

**Published:** 2022-02-24

**Authors:** Hazem Almhanna, Nabeel Abd Murad Al-Mamoori, Hassan Hachim Naser

**Affiliations:** 1 Department of Anatomy, Histology and Embryology, College of Veterinary Medicine, University of Al-Qadisiyah, Al-Qadisiyah, Iraq; 2 Department of Microbiology, College of Veterinary Medicine, University of Al-Qadisiyah, Al-Qadisiyah, Iraq

**Keywords:** angiotensin-converting enzyme 2, expression, rats, real-time polymerase chain reactions, severe acute respiratory syndrome coronavirus 2

## Abstract

**Background and Aim:**

Angiotensin-converting enzyme 2 (ACE2) is expressed and plays functional and physiological roles in different tissues of the body. This study aimed to distinguish the levels of expression of ACE2 in the lung tissue at different ages of rats.

**Materials and Methods:**

In this study, 18 male rats were used and divided into three groups according to age. Real-time quantitative polymerase chain reaction (RT-qPCR) was conducted to determine the levels of the quantification of eosinophil cationic protein mRNA transcript. In addition, tissue specimens of the lung were stained with routine hematoxylin and eosin stains.

**Results:**

This study confirmed that RT-qPCR amplification plots of ACE2 gene exhibited clearly expression of the lung tissue of rats in the different groups and there are strong different threshold cycles numbers according to the age at 2 weeks, 2 months, and 6-8 months. Consequently, the expression of ACE2 was completely different between groups depending on the age of the rats. The RT-qPCR results showed that the older animal group (age of 6-8 months) had a significantly higher expression of ACE2 than the other animal groups (ages of 2 weeks and 2 months). In the same way, the second group (age of 2 months) had a significantly higher expression of ACE2 than the first group (age of 2 weeks). This study confirmed that the ACE2 expression is influenced by the age of rats.

**Conclusion:**

This study concluded that the expression of the ACE2 receptor of coronavirus disease 2019 would be different according to the age of rats, and this result suggested that expression of ACE2 in lung tissue could determine infection and pathogenesis of COVID-19 during different ages of rats or some individual differences.

## Introduction

Angiotensin-converting enzyme 2 (ACE2) is an enzyme found in the cell membrane of different tissues of the body. Extensive research has identified ACE2 in the tissues of the intestine, heart, kidney, gallbladder, lung, and testis [1-5]. Structurally, ACE2 is a glycoprotein and has some similarities with the ACE [6]. ACE2 is considered a type I integral glycoprotein found in the cell membrane of most cells of the body and has six potential N-glycosylation sites [7]. In addition, it is a carboxypeptidase that cleaves a single hydrophobic/basic residue that extends from the C-terminus of its substrates [8].

The ACE2 is structurally considered vasoconstrictor and vasodilator peptide; therefore, it can be led to balance of the blood pressure of the body by regulating the heart and kidney functions [9,10]. Much evidence explained that ACE2 has a physiological and pathological influence on the renal, respiratory, and cardiovascular systems, such as acute respiratory distress syndrome [11].

More studies were noticed that using the ACE2 protects the kidneys of mice which were suffered experimentally diabetic nephropathy and mediated treatment pathway of the kidney injury. In addition, it is considered an important determinant of diabetic nephropathy [12,13]. It may regulate the effects and levels of angiotensin 2 in the kidneys [14] and regularly protect the heart and kidney functions [15]. Therefore, ACE2 is correlated with cardiac, vascular, and renal dysfunctions [16].

Importantly, severe acute respiratory syndrome coronavirus 2 (SARS-CoV-2) infection in cats and ferrets is related to the distribution of ACE2, a receptor found in the respiratory system, including the lung tissue, serous cells of the tracheobronchial submucosal glands in cats, and type II pneumocytes in ferrets [17-19].

Recent reports and articles about the coronavirus disease 2019 (COVID-19) pandemic have established that this disease is caused by SARS-CoV-2, which uses ACE2 receptors in the respiratory system [20-22]. Significantly, a structural analysis recognized residues in SARS-CoV-2 that is essential for ACE2 binding [23]. The entry point of SARS-CoV-2 into pneumocytes and the epithelium of the bronchus and trachea is the ACE2 receptor; consequently, the spikes in the capsule of the coronavirus bind to ACE2 before causing infection and viremia [24,25].

Several articles have characterized the infection, symptoms, and clinical signs of COVID-19 between children and adults; however, the majority of these studies confirmed that children could be infected with COVID-19, but the clinical signs are less severe than those in adults [26,27].

Correspondingly, researchers reported that the mortality and morbidity of COVID-19 were very low in children and young adults compared with those in adults [28,29]. Accordingly, this study hypothesized that in COVID-19, the expression of the ACE2 receptor in the respiratory system would normally increase with the increasing age of the rat. Wistar rats were chosen for this experiment as animal models for more investigations about this disease.

We hypothesized that the age of rats could influence the levels of expression and development of ACE2 in lung tissue. This study aimed to evaluate the ACE2 expression in the lung tissue of rats at different ages, which may provide answers regarding some speculations about COVID-19 infections between different ages of people.

## Materials and Methods

### Ethical approval

The Animals and Ethics Committee of the University of Kufa approved to the study to collect samples from rats (Approval no. 1192).

### Study period and location

This study was conducted from 10^th^ November 2020 to 1^st^ July 2021 in the Laboratory of College of Veterinary Medicine, University of Al-Qadisiyah.

### Samples

Eighteen male Wistar rats were obtained from the Animal House of the Faculty of Veterinary Medicine, University of Qadisiyah, and were divided into three groups according to age. The healthy rats were chosen for this study to avoid any physiological or biological changes in the organs and tissue of rats. The first group was 2 weeks, the second group was 2 months, and the third group was 6-8 months.

Rats were anesthetized before being killed by chloroform through the artificial inspiration for 2 min. Animals were killed, and samples of lung tissue were collected for each group. The samples were divided into two group samples, the first group samples were stored in 10% neutral buffered formalin (NBF) for histology study and second group samples were stored in AccuZol™ Total RNA Extraction Reagent (Bioneer, Korea) for real-time quantitative polymerase chain reaction (RT-qPCR).

### Extraction method of RNA

The mRNA of tissue was extracted using the AccuZol® reagent kit (Bioneer) according to the manufacturer’s instructions. Briefly, 200 mg of tissue was weighed and placed in a 1.5 mL Eppendorf Tube, and 1 mL ofAccuZol reagent (Bioneer) was added. Subsequently, 200 μL of chloroform (Chem-Lab, Belgium) was added to each tube and shaken robustly for 15 seconds. Then, the mixture was incubated on ice for 5 min and centrifuged (Thermo Fisher Scientific, Germany) at 14,000× g at 4°C for 15 min. The pellets were neglected, and the supernatant was removed in the Eppendorf tube (Abdos LabTech, India) and added with 500 μL of isopropanol. Then, the mixture was mixed by inverting the tube 4-5 times and incubated at 4°C for 10 min. The samples were centrifuged at 14,000× g at 4°C for 10 min. The supernatant was poured off, added 1 mL of 80% ethanol, and mixed again using a vortex. Subsequently, the samples were recentrifuged at 14,000× g at 4°C for 10 min. The supernatant was neglected, and the RNA pellet yield was exposed to air for drying. Finally, the RNA pellets were loaded with 50 μL of diethylpyrocarbonate water (Bioneer) to each sample to dissolve them; then, these were stored in a freezer at −20°C. A NanoDrop spectrophotometer (Thermo Fisher Scientific, USA) was used to evaluate the RNA concentration.

### DNase I treatment and cDNA synthesis

The samples were treated with DNase I enzyme to digest the DNA and extract the RNA using the DNase I enzyme kit (Promega Corporation, USA) according to the manufacturer’s instructions. Then, cDNA from the extracted RNA was synthesized using the AccuPower® RocktScript RT PreMix kit according to the manufacturer’s instructions. The RNA was translated into cDNA in a thermocycler (Bio-Rad, USA) under the following conditions: cDNA synthesis (RT step) was conducted at 50°C for 1 h, and heat inactivation was conducted at 95°C for 5 min.

### RT-qPCR

RT-qPCR was conducted to determine the levels of the quantification of the eosinophil cationic protein mRNA transcript, and the RT-qPCR system (Bio-Rad Laboratories Inc., USA) was used to assess the RT-qPCR reactions for the ACE2 target gene. The SYBR Green qPCR Master Mix was applied to determine the amplification of the ACE2 gene expression and glyceraldehyde-3-phosphate dehydrogenase (GAPDH) housekeeping gene for the normalization of gene expression. Primers were designed using the Primer3Plus (Table-1 lists the primer sequences, which were made by Scientific Researcher Co. Ltd., Iraq).

**Table 1:** This table showed the RT-qPCR primers with their sequence.

Primer	Sequence (5’-3’)		Amplicon size	GenBank accession no.
ACE2	F	GCCGTTGGAGAAATCATGTCAC	147bp	NM_001012006.1
	R	TGGCAGCGTTCCAACAATTG		
GAPDH	F	ATGCCCCCATGTTTGTGATG	136bp	NM_001012006.1
	R	TCCACGATGCCAAAGTTGTC		

RT-qPCR=Real-time quantitative polymerase chain reaction, ACE2=Angiotensin-converting enzyme 2, GAPDH=Glyceraldehyde-3-phosphate dehydrogenase

The qPCR Master Mix was prepared for the specific target gene and the GAPDH housekeeping gene according to the manufacturer’s instructions (AccuPower® 2XGreen Star qPCR Master Mix kit; Bioneer). Furthermore, the thermocycler conditions were applied according to Table-2. The gene expression target was analyzed using the 2-∆∆CT Livak method [30].

**Table 2:** This table shows the RT-qPCR thermocycler conditions.

RT-qPCR step	Temperature	Time	Repeat cycle
cDNA Step	50°C	1 hour	1
Denaturation	95°C	20 sec	45
Annealing\Extension Detection(scan)	60°C	30 sec	
Melting	60-95°C	0.5 sec	1

### Histological process of tissue

Tissue specimens were fixed in 10% formalin (NBF) and washed with tap water for 2 h; subsequently, these specimens underwent a series of ethyl alcohol washes and then with xylene (twice) for 2 min for each wash. Next, tissues were embedded in paraffin, and tissue sections were prepared. Paraffin was removed by xylene and rehydrated, and sections were stained using routine hematoxylin and eosin stains. The sections were examined under light microscopy (Olympus, Japan), and digital images were obtained for each section using 10× [31].

### Statistical analysis

The RT-qPCR raw data were analyzed using Excel 2019 (Microsoft, USA). The means and standard errors of the gene for three groups were determined, and the ACE2 expression in different ages was assessed using one-way single-factor analysis of variance.

## Results

**Figure-1:**
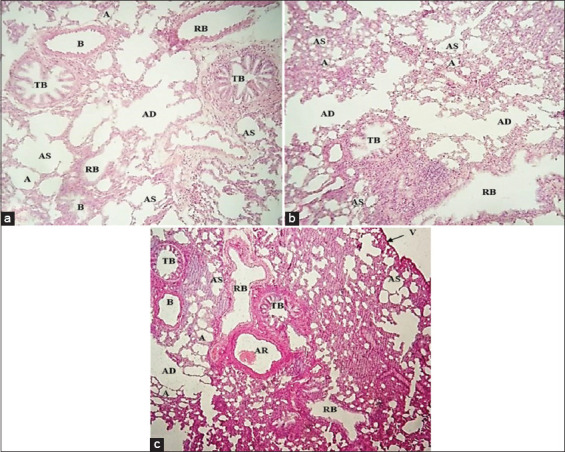
These images illustrated the histological structure of the lung tissue of rats for different groups. Indicator (a) is a first group and age (2 weeks), (b) is a second group and age (2 months), and (c) is a third group and age (6-8 months). AS=Alveolar sac, B=Bronchioles, TB=Terminal bronchiole, RB=Respiratory bronchiole, AD=Alveolar duct, A=Alveoli, AR=Artery, V=Visceral pleura (H and E, staining 100×).

In this study, three groups of rats according to age (first group, 2 weeks; second group, 2 months; and third group, 6-8 months) were used to determine the levels of expression of ACE2 in the lung tissue. The fresh samples were used for histology and RT-qPCR study. Subsequently, the anatomical inspection showed that the right lung was divided into four lobes and the left lung had one lobe and was undivided. Histologically, the lung was enclosed by a single layer of squamous epithelial cells overlying connective tissue (visceral pleura). The lung parenchyma showed bronchioles of different sizes and shapes (terminal and respiratory bronchioles). The epithelium lining of the bronchiole gradients was changed from pseudostratified columnar to simple columnar. The respiratory bronchioles are also divided into alveolar ducts, which lead to the alveolar sacs and alveoli (Figure-1).

**Figure-2:**
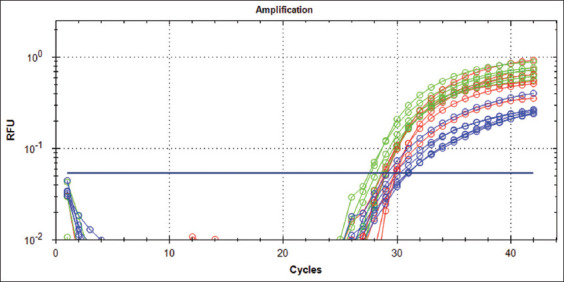
This figure presented the RT-qPCR amplification plots of the angiotensin-converting enzyme-2 gene of the lung tissue of rats for different groups. The blue plots (first group), the red plots (second group), and the green plots (third group).

The RT-qPCR amplification plots of ACE2 gene of the lung tissue were precisely observable threshold cycles (Ct) numbers of expression which were different between groups of rats according to the age at 2 weeks, 2 months, and 6-8 months (range, C_T_ 30.2- C_T_ 32.59, C_T_ 29.36- C_T_ 30.81, and C_T_ 28.17- C_T_ 29.56, respectively (Figure-2).

**Figure-3:**
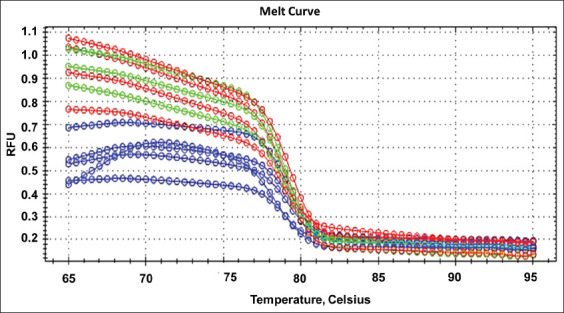
This figure displayed the RT-qPCR melting curve of the angiotensin-converting enzyme-2 gene of the lung tissue of rats for different groups. The blue plots (first group), the red plots (second group), and the green plots (third group).

**Figure-4:**
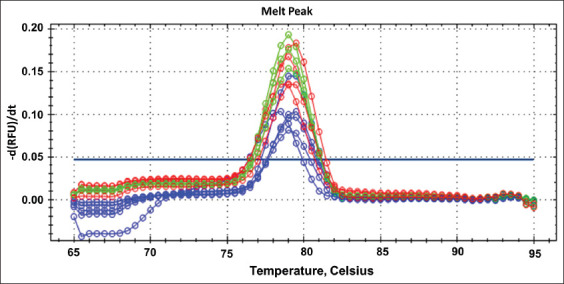
This figure exhibited RT-qPCR melting peak of the angiotensin-converting enzyme-2 gene for the rat lung tissue of experimental groups. The blue plots (First group), the red plots (second group), and the green plots (third group) showed that RT-qPCR melting temperature was 79-80°C.

The specificity of RT-qPCR primer was tested by melting peak analysis which was shown a highly specific without any non-specific products amplification; moreover, all experimental specimens had a melting peak ranging from 79°C to 80°C (Figures-2 and 3). The RT-qPCR efficiency was assessed using the cDNA standard curve for experimental samples and was shown to be 108% (Figure-4).

**Figure-5:**
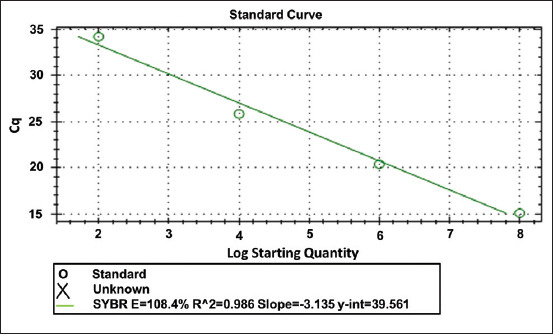
This figure displayed the RT-qPCR standard curve of angiotensin-converting enzyme-2 gene for the rat lung tissue of experimental groups and showed that RT-qPCR efficiency was 108%.

**Figure-6:**
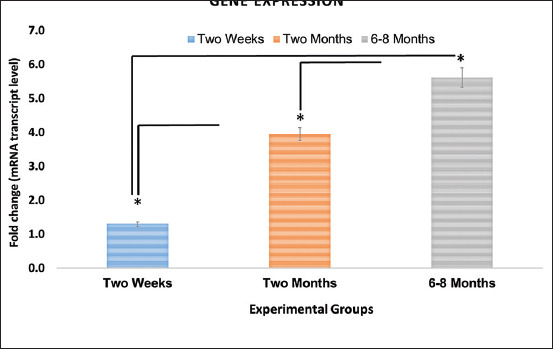
This histogram showed the comparison of the total gene expression between groups. Graph showing total expression of angiotensin-converting enzyme-2 that expressed in lung tissue of rats at a different stage of the age. Each column represents the group and age of the rats. Three groups (p=0.05) displayed significantly higher between groups. *Indicates significant values derived using analysis of variance (single factor).

Our findings showed that the ACE2 expression was significantly different between groups of rats depending on their age. The RT-qPCR results showed that the older animal group (age of 6-8 months) had a significantly higher expression of ACE2 than the other animal groups (ages of 2 weeks and 2 months). In the same way, the second group (age of 2 months) had a significantly higher expression of ACE2 than the first group (age of 2 weeks) (Table-3 and Figures-5 and 6).

**Table 3:** This table displayed values of gene expression and housekeeping gene which were analyzed using 2-∆∆CT Livak method. ACE2=Angiotensin-converting enzyme 2.

Experimental groups	CT ACE2	CT GAPDH	ACT test	∆CT control	∆∆CT control	Fold change (2^∆CT)	Mean and St. error for Exp. groups
A	31.77	25.39	6.38	7.37	–0.99	1.986	1.297±0.38
A	32.52	24.96	7.56	7.37	0.19	0.877	
A	30.2	24.29	5.91	7.37	–1.46	2.751	
A	32.59	23.26	9.33	7.37	1.96	0.257	
A	32.52	25.50	7.02	7.37	–0.35	1.275	
A	31.16	23.14	8.02	7.37	0.65	0.637	
B	29.36	24.20	5.16	7.37	–2.21	4.627	3.947±0.57
B	30.81	24.53	6.28	7.37	–1.09	2.129	
B	30.39	24.65	5.74	7.37	–1.63	3.095	
B	29.59	24.45	5.14	7.37	–2.23	4.691	
B	30.3	24.59	5.71	7.37	–1.66	3.160	
B	29.42	24.63	4.79	7.37	–2.58	5.979	
C	28.8	24.12	4.68	7.37	–2.69	6.453	5.616±0.74
C	28.17	22.55	5.62	7.37	–1.75	3.364	
C	29.18	24.60	4.58	7.37	–2.79	6.916	
C	28.24	23.73	4.51	7.37	–2.86	7.260	
C	29.51	23.84	5.67	7.37	–1.70	3.249	
C	29.56	24.88	4.68	7.37	–2.69	6.453	
Mean A	31.79	24.42	7.37				

These findings confirmed that the expression of ACE2 in the lung tissue would be different depending on the age of animals. For this reason, it seemed that immature animals would be less infected with COVID-19 compared with mature ones.

## Discussion

The ACE2 is a cellular transmembrane glycoprotein and has an extensive homolog to carboxypeptidase ACE. Moreover, it ends with non-catalytic extracellular and cytosolic domains, but lacks active dipeptidyl carboxypeptidase catalytic domains [32-34].

Several researchers described that ACE2 is found in various cells and tissues in rodents, with high expression in the heart, lymphoid tissues, and digestive and respiratory systems and low expression in the central nervous system [35-38]. Our results confirmed that ACE2 is expressed in the lung tissue of rats. ACE2 has synchronized physiological and biological functions between cells; therefore, its expression levels would allow for homeostatic regulation of circulating angiotensin II. It is attached to the cell membrane in endothelial cells and lung tissue and could be shed from the cell surface and regulate other physiological functions [39,40]. This study determined the expression of the ACE2 in the lung tissue, which regulates the function of epithelial cells and pneumocytes.

Our study determined that the transcriptome expression of ACE2 was dependent on the age of rats and there were differences between mature and immature ones. Other researchers have confirmed that the ACE2 receptor is considered the entry point of important viruses, such as SARS-CoV and SARS-CoV-2 [41-44]. Therefore, the pathogenesis of an infection could be determined depending on the expression of ACE2.

Many studies have indicated that SARS-CoV and SARS-CoV-2 infect adults and elderly people more than children; thus, the clinical signs are less severe than those in adults [45-48]. Taken together, these previous studies are in agreement with our results, which confirmed that the level of expression of ACE is significantly higher in mature rats than in immature rats and younger.

The high level of expression of ACE2 in the respiratory system may be used to predict the occurrence of COVID-19 infection and the prognosis of individuals infected with the virus. Moreover, the age of people would be restricted the distribution and expression of ACE2 receptor tissue in the respiratory system, which is being the entry point of the virus; thus, the level of the receptor would be influenced by the prognosis’s COVID-19 and detected the severity of infection, mortality, and morbidity [49,50]. Therefore, these studies and our results confirmed that the expression of ACE2 is different between ages and other factors. For these reasons, SARS-CoV-2 infection would also be different between people.

The level of expression of ACE2 would determine the severity of SARS-CoV-2 infections after exposure to the virus [51,52], which could be expected the severe clinical signs of infected people, and percentage of morbidity and mortality. Overall, this result explained that the age of rats would influence the expression levels of ACE2 in lung tissue, which may predict the type of infection, that is, COVID-19 and SARS-CoV infections.

## Conclusion

This study shows that the ACE2 receptors in the lung tissue are differentially expressed according to the age of rats; therefore, the age of rats affects the expression levels of ACE2. This result may provide some clarifications regarding the coronavirus infection and other infectious diseases concerning receptors in the respiratory system. Moreover, the severity of the coronavirus disease would be determined depending on the expression of ACE2 in the respiratory system; therefore, more studies should be focused on these receptors in the upper and lower respiratory system.

## Authors’ Contributions

HA: Designed the experiment and reviewed the manuscript. HHN: Conducted the study, performed the experimental laboratory work, and revised the manuscript. HA: Data analysis, project advisor, and reviewed the manuscript. NAMA: Conducted the literature review, interpreted the data, and drafted the manuscript. All authors have revised and approved the final manuscript.
